# Expert consensus on the management of systemic sclerosis-associated interstitial lung disease

**DOI:** 10.1186/s12931-022-02292-3

**Published:** 2023-01-09

**Authors:** Franck F. Rahaghi, Vivien M. Hsu, Robert J. Kaner, Maureen D. Mayes, Ivan O. Rosas, Rajan Saggar, Virginia D. Steen, Mary E. Strek, Elana J. Bernstein, Nitin Bhatt, Flavia V. Castelino, Lorinda Chung, Robyn T. Domsic, Kevin R. Flaherty, Nishant Gupta, Bashar Kahaleh, Fernando J. Martinez, Lee E. Morrow, Teng Moua, Nina Patel, Oksana A. Shlobin, Brian D. Southern, Elizabeth R. Volkmann, Dinesh Khanna

**Affiliations:** 1 Respiratory Center, Cleveland Clinic Florida, 2950 Cleveland Clinic Blvd, Weston, FL 33331 USA; 2 Rutgers-RWJ Medical School, New Brunswick, NJ USA; 3 Weill Cornell Medicine, New York, NY USA; 4 University of Texas, Houston, TX USA; 5 Brigham and Women’s Hospital, Boston, MA USA; 6 University of California Los Angeles, Los Angeles, CA USA; 7 Georgetown University, Washington, D.C USA; 8 University of Chicago, Chicago, IL USA; 9 Columbia University Irving Medical Center, New York, NY USA; 10 Ohio State University, Columbus, OH USA; 11 Harvard Medical School, Boston, MA USA; 12 Stanford University School of Medicine and Palo Alto VA Health Care System, Stanford, CA USA; 13 University of Pittsburgh Medical Center, Pittsburgh, PA USA; 14 University of Michigan Scleroderma Clinic, Ann Arbor, MI 48105 USA; 15 University of Cincinnati, Cincinnati, OH USA; 16 University of Toledo Medical Center, Toledo, OH USA; 17 Creighton University, Omaha, NE USA; 18 Mayo Clinic, Rochester, MN USA; 19 Present Address: Boehringer Ingelheim Pharmaceuticals Inc, Ridgefield, CT USA; 20 Inova Fairfax Hospital, Falls Church, VA USA; 21 Cleveland Clinic, Cleveland, OH USA

**Keywords:** Autoimmune diseases, Connective tissue diseases, Pulmonary fibrosis, Drug therapy, Algorithms

## Abstract

**Background:**

Systemic sclerosis (SSc) is a rare, complex, connective tissue disorder. Interstitial lung disease (ILD) is common in SSc, occurring in 35–52% of patients and accounting for 20–40% of mortality. Evolution of therapeutic options has resulted in a lack of consensus on how to manage this condition. This Delphi study was initiated to develop consensus recommendations based on expert physician insights regarding screening, progression, treatment criteria, monitoring of response, and the role of recent therapeutic advances with antifibrotics and immunosuppressants in patients with SSc-ILD.

**Methods:**

A modified Delphi process was completed by pulmonologists (n = 13) and rheumatologists (n = 12) with expertise in the management of patients with SSc-ILD. Panelists rated their agreement with each statement on a Likert scale from − 5 (complete disagreement) to + 5 (complete agreement). Consensus was predefined as a mean Likert scale score of ≤  − 2.5 or ≥  + 2.5 with a standard deviation not crossing zero.

**Results:**

Panelists recommended that all patients with SSc be screened for ILD by chest auscultation, spirometry with diffusing capacity of the lungs for carbon monoxide, high-resolution computed tomography (HRCT), and/or autoantibody testing. Treatment decisions were influenced by baseline and changes in pulmonary function tests, extent of ILD on HRCT, duration and degree of dyspnea, presence of pulmonary hypertension, and potential contribution of reflux. Treatment success was defined as stabilization or improvement of signs or symptoms of ILD and functional status. Mycophenolate mofetil was identified as the initial treatment of choice. Experts considered nintedanib a therapeutic option in patients with progressive fibrotic ILD despite immunosuppressive therapy or patients contraindicated/unable to tolerate immunotherapy. Concomitant use of nintedanib with MMF/cyclophosphamide can be considered in patients with advanced disease at initial presentation, aggressive ILD, or significant disease progression. Although limited consensus was achieved on the use of tocilizumab, the experts considered it a therapeutic option for patients with early SSc and ILD with elevated acute-phase reactants.

**Conclusions:**

This modified Delphi study generated consensus recommendations for management of patients with SSc-ILD in a real-world setting. Findings from this study provide a management algorithm that will be helpful for treating patients with SSc-ILD and addresses a significant unmet need.

**Supplementary Information:**

The online version contains supplementary material available at 10.1186/s12931-022-02292-3.

## Background

Systemic sclerosis (SSc) is a rare, complex connective tissue disease of unknown etiology characterized by microvascular damage, dysregulation of innate and adaptive immunity, and generalized fibrosis in the skin and multiple internal organs [1–3]. It can target many organ systems, including the skin, lungs, heart, blood vessels, kidneys, gastrointestinal tract, and musculoskeletal system [2, 3]. While the pathogenesis of SSc is not well understood, hallmarks include inflammation, vasculopathy, and fibroblast dysfunction. Risk factors associated with the development of and/or progression of ILD include male sex, African-American race, diffuse cutaneous SSc (dcSSc), presence of anti-Scl-70/anti-topoisomerase I antibodies, and cardiac involvement [4–6].

Interstitial lung disease (ILD) occurs in 35–52% of patients with SSc and accounts for 20–40% of mortality [7–10]. The risk of developing ILD is greatest early in the course of SSc, and timely detection is important for monitoring progression and informing therapeutic decision-making [4, 11]. A European consensus statement identified chest high-resolution computed tomography (HRCT) as the primary diagnostic tool for SSc-ILD, supported by pulmonary function tests (PFTs) for screening and diagnosis [12]. Declining values for forced vital capacity (FVC) and diffusing capacity of the lungs for carbon monoxide (DL_CO_) can suggest progression of ILD [1, 4, 13].

The European League Against Rheumatism (EULAR) guidelines for the management of SSc recommend cyclophosphamide (CYC) for the treatment of SSc-ILD. Hematopoietic stem cell transplantation may also be considered in patients with rapidly progressive SSc at risk of organ failure, although careful selection of patients is required due to a high risk of treatment-related side effects and mortality [14–16]. Mycophenolate mofetil (MMF) provides similar efficacy to CYC and is associated with less toxicity [17–21]. For patients who do not respond to treatment, lung transplantation may be a life-saving option [22, 23].

Antifibrotic drugs represent another class that may be considered a potential treatment option for SSc-ILD based on their efficacy in idiopathic pulmonary fibrosis (IPF), a condition with pathogenic similarities. Nintedanib has been approved for the treatment of SSc-ILD and chronic fibrosing ILDs with a progressive phenotype based on the results of the SENSCIS and INBUILD trials respectively [24–26]; pirfenidone is currently under evaluation [27, 28]. In addition, tocilizumab (TCZ), an inhibitor of interleukin-6, has recently been approved for the treatment of SSc-ILD based on the results of the focuSSced trial [29, 30].

There is a growing desire among pulmonologists and rheumatologists to identify screening, detection, and monitoring strategies that are clinically meaningful and improve patient outcomes. The objective of this Delphi study was to develop consensus recommendations based on expert physician insights regarding screening, disease progression, treatment criteria, monitoring of therapeutic response, and the potential future role of antifibrotics in the treatment paradigm for patients with SSc-ILD.

## Methods

This modified Delphi process was conceived and initiated by the lead and corresponding authors (FFR and DK). As moderators, FFR and DK worked with an advisory board of pulmonologists and rheumatologists with experience in treating SSc-ILD, and Boehringer Ingelheim Pharmaceuticals Inc., to determine the panel selection criteria and identify potential members.

**Fig. 1:**
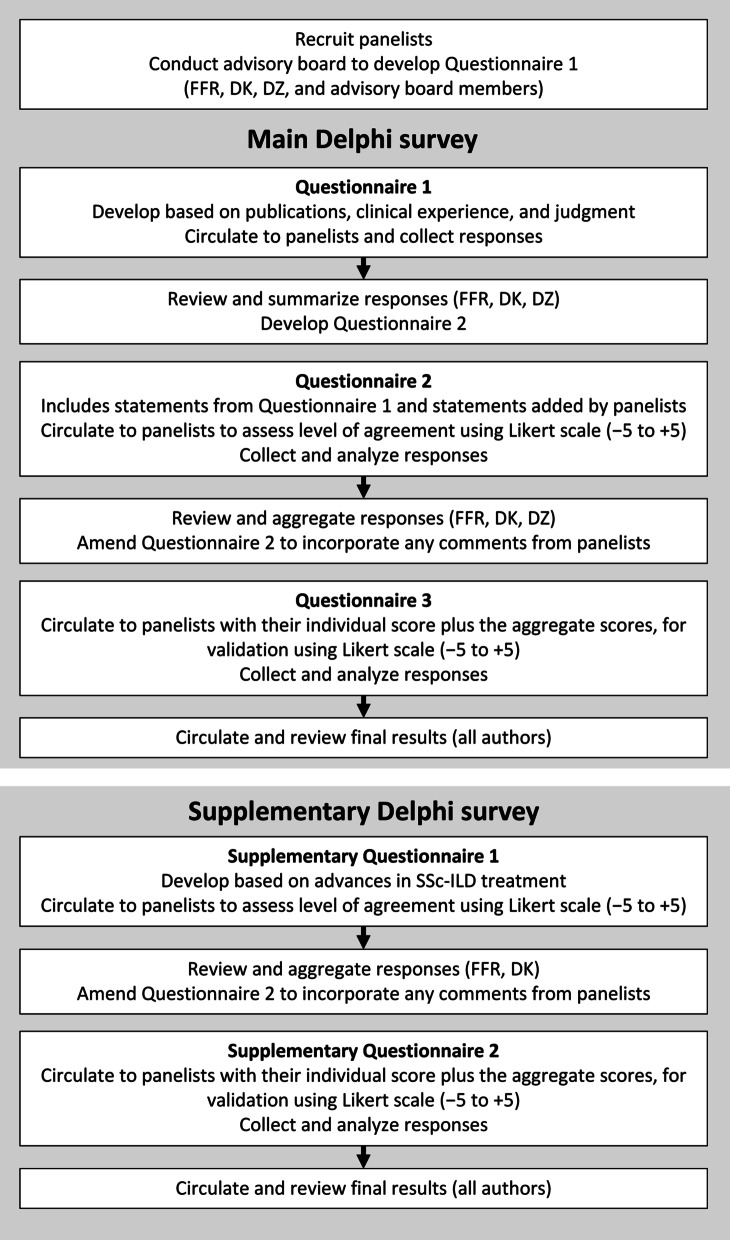
The Delphi process employed for both the first and second Delphi analyses. *SSc-ILD* systemic sclerosis-associated interstitial lung disease

The modified Delphi process followed in this study was as follows (Fig. 1):

1. The moderators and advisory board members developed Questionnaire 1 based on current clinical practice, a review of the literature, and clinical experience. This topically organized questionnaire included multiple statements relevant to screening, treatment criteria, and the potential role for antifibrotic drugs in patients with SSc-ILD. These topics were maintained through all three questionnaires. The Questionnaire was circulated via an online survey platform (Surveygizmo.com) to the Delphi panelists, who provided their independent comments for each statement and added additional statements at their discretion.
2. Following review of the aggregate anonymized responses to Questionnaire 1, the moderators developed Questionnaire 2, which incorporated both the initial and additional statements from the panelists. Questionnaire 2 was circulated using the same online survey platform and the panelists rated each statement using a Likert scale ranging from –5 (strongly disagree) to + 5 (strongly agree). Consensus was defined prospectively as a mean rating of ≤ –2.5 or ≥  + 2.5, with a standard deviation (SD) that did not cross zero.
3. The moderators refined the statements based on the aggregate results from Questionnaire 2 to create the 152-statement Questionnaire 3, again circulated using the online survey platform. To promote consensus ‘for’ or ‘against’ each statement, panelists also received separately their individual responses to Questionnaire 2 plus the panel’s aggregate results (mean and SD). Additional questions regarding the role of nintedanib in the treatment of SSc-ILD were shared with the panel following publication of the SENSCIS trial results.
4. The aggregate results of Questionnaire 3 were circulated to the panelists for final review and comment (see Additional file 1: Table S1 for details).
5. Following publication of the INBUILD, RELIEF, and focuSSced trial results [25, 27, 29], a supplementary Delphi survey comprising two questionnaires focusing on the current treatment paradigm was conducted. The same approach was used as described above.

Panelists’ anonymity was ensured throughout the study to prevent bias induced by influential clinicians and help ensure that all panelists were comfortable offering their opinions freely. Panelists were encouraged to provide feedback on the validity, specificity, and content of the items under consideration.

## Results

The Delphi process was initiated in 2018 with a panel comprising 25 physicians (Table 1). The panel included 13 pulmonologists and 12 rheumatologists practicing predominately in academic centers (n = 24), hospital-based clinics (n = 3), and Veterans Administration (n = 2). Some panelists practiced across multiple care settings. Their collective experience of seeing patients with SSc-ILD was 16.68 ± 9.68 (mean ± SD) years, treating 80.04 ± 73.23 patients with SSc-ILD in the last year (Table 1). The panel reached consensus on 109 of the 239 statements (45.6%); 98 statements reached consensus ‘for’ and 11 reached consensus ‘against.’ A list of all questions and results from Questionnaire 3 and Supplementary Questionnaire 2 can be viewed in Additional file 1: Tables S1 and S2.

**Table 1:** Characteristics of the Delphi panelists

Characteristic	Number of panelists	Mean ± SD
Specialty		
Pulmonology	13	
Rheumatology	12	
Experience treating SSc-ILD (n = 25)		16.68 ± 9.68
≤ 10 years	6	
11–20 years	14	
> 20 years	5	
Patients with SSc-ILD treated in entire career (n = 24)		369.58 ± 341.74
≤ 500	21	
501–1000	2	
> 1000	1	
Patients with SSc-ILD treated last year (n = 23)		80.04 ± 73.23
≤ 50	11	
51–150	10	
> 150	2	

*SD* standard deviation, *SSc-ILD* systemic sclerosis-associated interstitial lung disease

### Screening

**Fig. 2:**
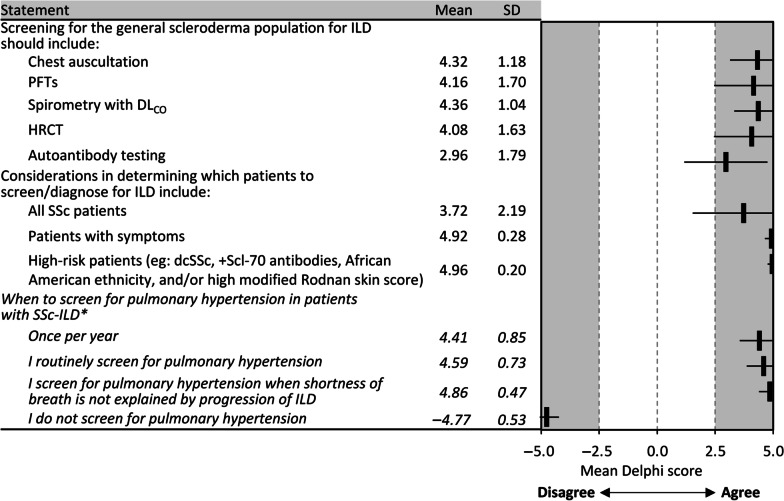
Consensus recommendations for screening criteria for SSc-ILD. *Data from the 2022 supplementary Delphi. *dcSSc* diffuse cutaneous systemic sclerosis, *DL*_*CO*_ diffusing capacity of the lungs for carbon monoxide, *HRCT* high-resolution computed tomography, *ILD* interstitial lung disease, *PFT* pulmonary function test, *SD* standard deviation, *SSc* systemic sclerosis

Panelists recommended the use of chest auscultation, full PFTs, spirometry with DL_CO_, HRCT, and autoantibody testing to screen patients with SSc for ILD (Fig. 2, Additional file 1: Table S1). The consensus was that all patients with SSc should be screened, with greater agreement for patients with respiratory symptoms and those at high risk (e.g. dcSSc, positive for Scl-70 antibodies, African-American ethnicity, and/or a high modified Rodnan skin score [mRSS]). In addition, panelists recommended routinely screening for pulmonary hypertension (PH) in patients with SSc; the consensus to screen for this was even stronger when shortness of breath not explained by progression of ILD is observed.

### Treatment criteria

**Fig. 3:**
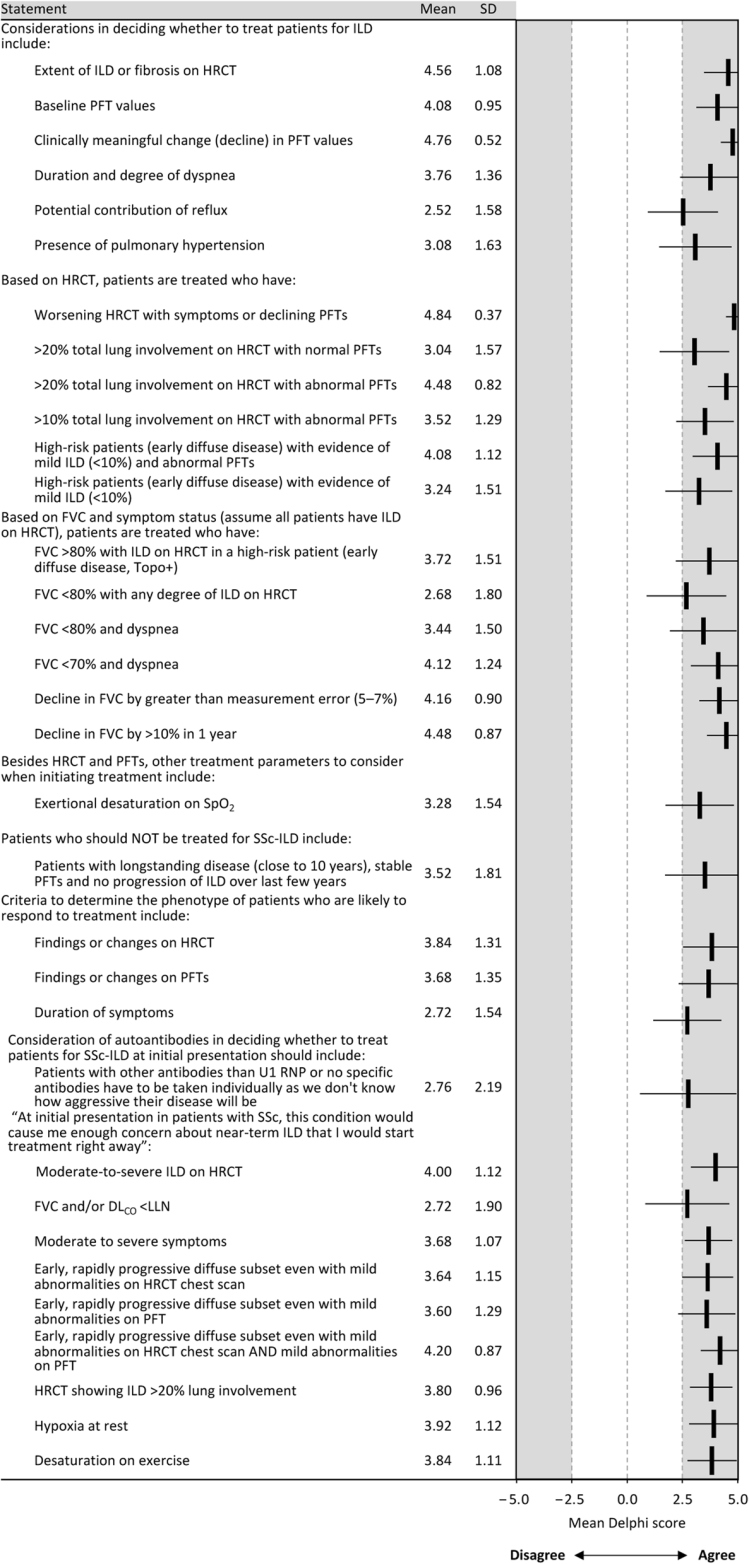
Consensus recommendations for SSc-ILD treatment criteria. *dcSSc* diffuse cutaneous SSc, *DL*_*CO*_ diffusing capacity of the lungs for carbon monoxide, *FVC* forced vital capacity, *HRCT* high-resolution computed tomography, *ILD* interstitial lung disease, *LLN* lower limit of normal, *PFT* pulmonary function test, *RNP* ribonucleoprotein, *SD* standard deviation, *SpO*_*2*_ peripheral capillary oxygen saturation, *SSc* systemic sclerosis, *Topo* topoisomerase

Panelists reached consensus that their treatment decisions were influenced by baseline and clinically meaningful changes in PFT values, the extent of ILD or fibrosis on HRCT, the duration and degree of dyspnea, the presence of PH, and the potential contribution of reflux (Fig. 3). There was no consensus regarding the use of autoantibody status, age, presence of comorbidities, or duration of disease (Additional file 1: Table S1). The panelists recommended that treatment should be initiated in patients with abnormal or progressive findings on HRCT, FVC < 80%, or FVC > 80% if accompanied by ILD in a high-risk patient or by dyspnea, by a notable decline in FVC, or accompanied by peripheral capillary oxygen desaturation (SpO_2_) on exercise (Fig. 3). Moderate-to-severe ILD on HRCT (or > 20% lung involvement), FVC and/or DL_CO_ below the normal lower limit, moderate-to-severe symptoms, early rapidly progressive dcSSc (even if accompanied by only mild abnormalities on HRCT and/or PFTs), hypoxemia at rest, and desaturation on exercise were considered sufficiently concerning to warrant immediate treatment. The panel also reached consensus that patients with longstanding disease (close to 10 years), stable PFTs, and no progression of ILD over recent years should not be treated (Additional file 1: Table S1).

### Treatment sequencing

**Fig. 4:**
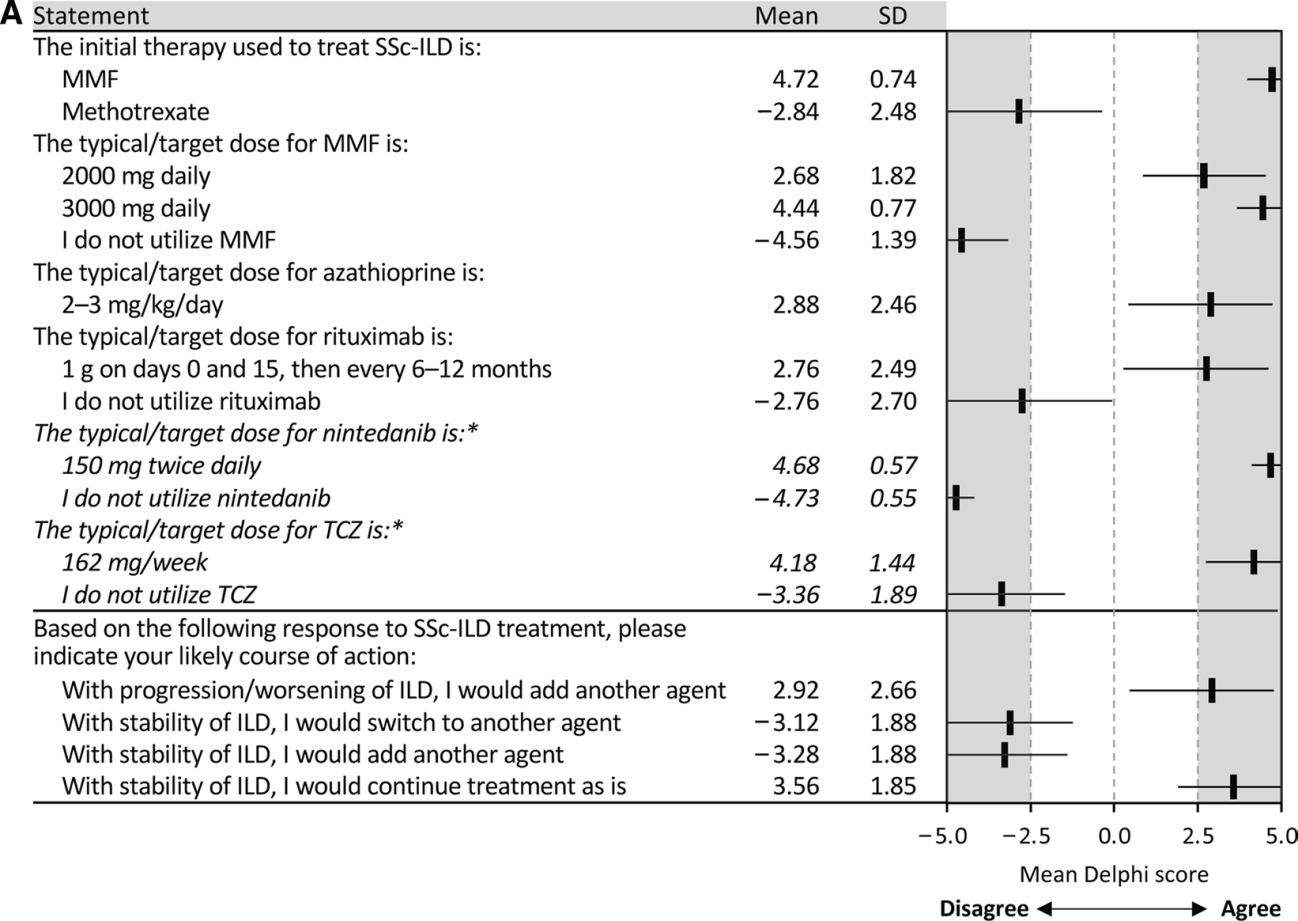

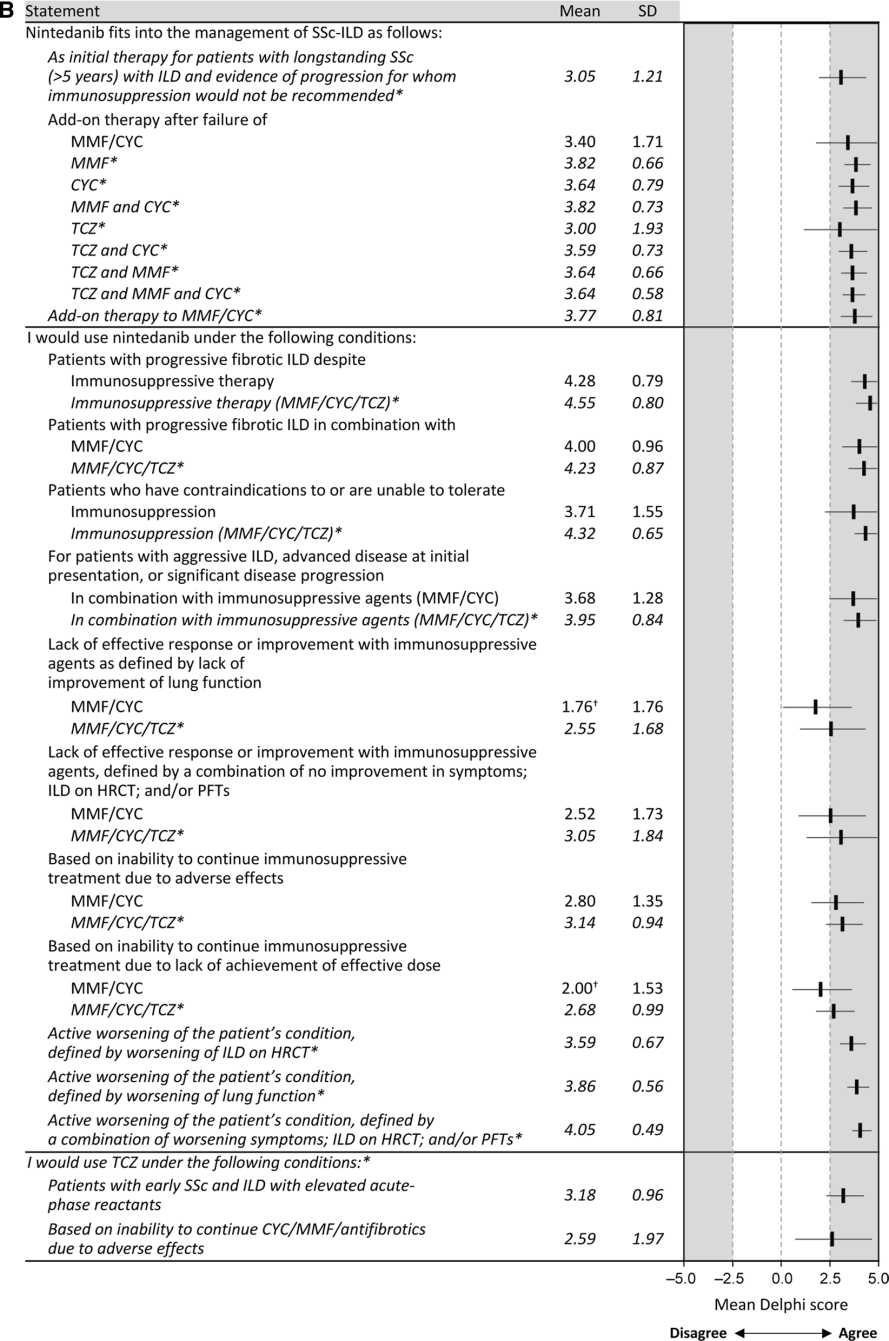
Consensus recommendations for SSc-ILD treatment paradigm. **A** Treatment dosage and next steps following treatment response. **B** Use of nintedanib and tocilizumab. *Data from the 2022 supplementary Delphi. ^†^Consensus was not reached in main Delphi analysis. *CYC* cyclophosphamide, *HRCT* high-resolution computed tomography, *ILD* interstitial lung disease, *MMF* mycophenolate mofetil, *PFT* pulmonary function test, *SD* standard deviation, *SSc* systemic sclerosis, *TCZ* tocilizumab

Panelists considered MMF as first-line therapy for patients with SSc-ILD at a target dose of 2000–3000 mg daily. Initial nintedanib therapy, at a target dose of 150 mg twice daily, was recommended for patients with longstanding (> 5 years) SSc with ILD and evidence of progression for whom immunosuppression would not be recommended. (Fig. 4A, Additional file 1: Table S2). It was also considered as add-on therapy to MMF or CYC and following failure of MMF, CYC, and/or TCZ. However, there was no consensus on the use of CYC, rituximab, azathioprine, prednisone, or TCZ as initial therapy, nor for the use of nintedanib as initial therapy in patients other than those previously described (Additional file 1: Table S1). There was consensus against utilizing methotrexate as initial therapy for patients with SSc-ILD, and there was no consensus on the duration of treatment.

### The potential role of antifibrotic drugs

When asked how the panel’s treatment choices for patients with SSc-ILD have changed since publication of the SENSCIS, INBUILD, and focuSSced trial results, 77% mentioned that they had increased their use of nintedanib or antifibrotics, while 45% mentioned that they now use or would consider using TCZ.

Antifibrotic drugs in general were considered potential therapeutic options based on a decline in PFTs and/or HRCT, and it was recommended that they should be used in combination with or after MMF/CYC. Following the results of the SENSCIS and INBUILD trials, the panelists reached consensus that nintedanib specifically was a therapeutic option for patients with progressive fibrotic ILD despite immunosuppressive therapy, both as monotherapy and in combination with MMF/CYC/TCZ, for patients with aggressive ILD (defined as relative FVC decline > 10% in 1 year), with advanced disease at initial presentation (FVC < 50%) in combination with MMF/CYC/TCZ, and for patients with contraindications to or who are unable to tolerate immunosuppression (MMF/CYC/TCZ). There was also consensus that nintedanib could be used to treat patients unable to continue with MMF, CYC, or TCZ due to adverse effects or lack of efficacy with the effective dose (Fig. 4B, Additional file 1: Table S2). The panelists’ consensus was that the decision to include nintedanib in the treatment regimen should be based on lack of an effective response to MMF/CYC/TCZ, defined by a combined lack of improvement in symptoms, HRCT, and/or PFT results, or an active worsening of the patient’s condition, defined by worsening of ILD on HRCT, worsening of lung function, or a combination of the two with worsening symptoms.

### The potential role of TCZ

Following the results of the focuSSced trial, the panelists agreed that TCZ should be considered in patients with early SSc and ILD with elevated acute-phase reactants. Use of TCZ in patients experiencing worsening on initial therapy did not achieve consensus by the whole group. They also considered TCZ an option for patients unable to continue CYC, MMF, or antifibrotics due to adverse effects. There was no consensus agreement on how TCZ fits into the management of SSc-ILD (Fig. 4B, Additional file 1: Table S2).

### Management of disease progression

**Fig. 5:**
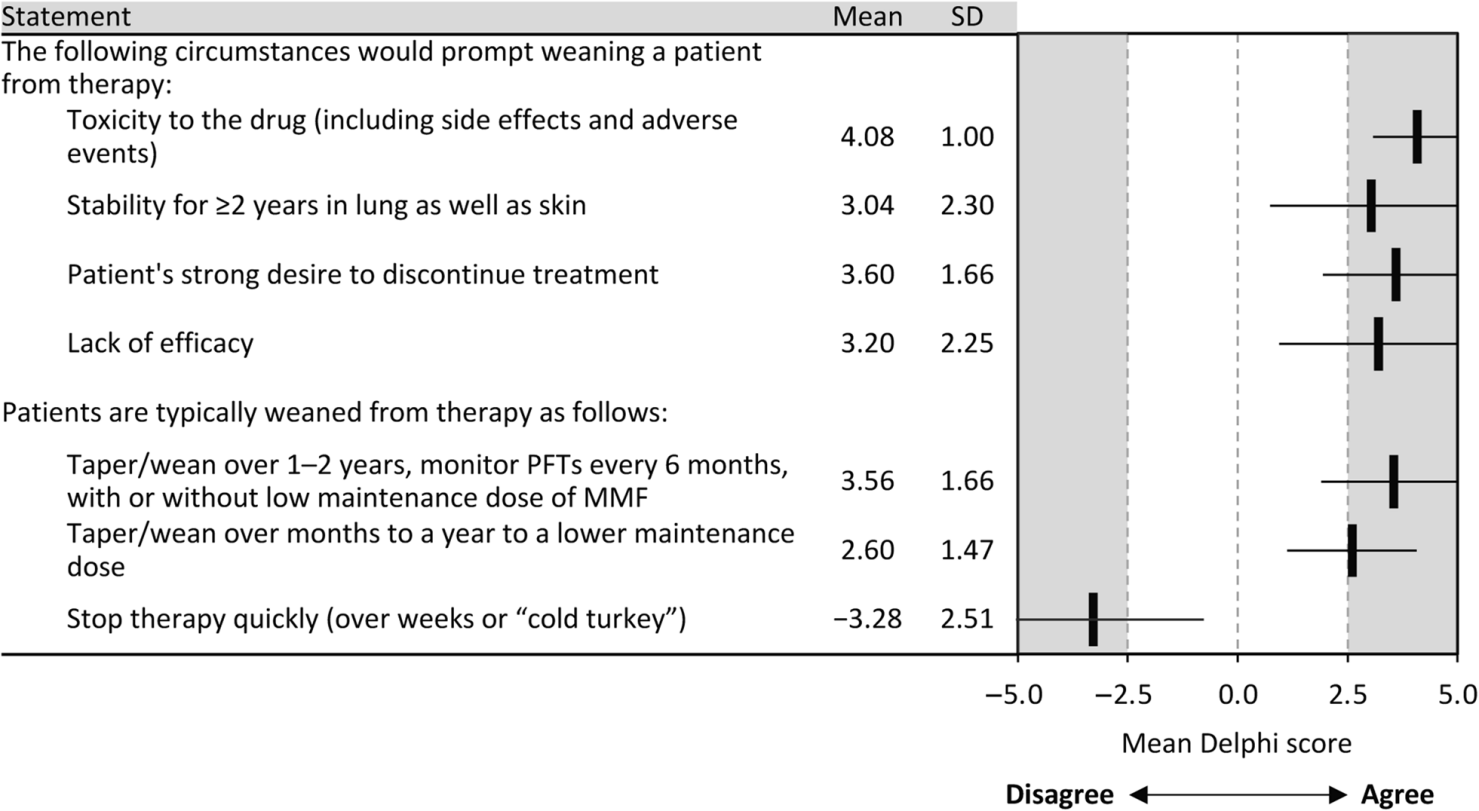
Consensus recommendations for duration of SSc-ILD treatment. *MMF* mycophenolate mofetil, *PFT* pulmonary function test, *SD* standard deviation, *SSc-ILD* systemic sclerosis-associated interstitial lung disease

Panelists reached consensus on the possibility of adding further agents for patients with progression/worsening of ILD, but did not reach consensus on switching to another agent or continuing therapy under these circumstances. Panelists agreed that patients should be weaned from therapy in cases of drug toxicity, stable lung and skin symptoms for ≥ 2 years, a patient's strong desire to discontinue treatment, or lack of efficacy (Fig. 5). Tapering/weaning therapy should take place over 1–2 years while monitoring PFTs every 6 months, with or without a low maintenance dose of MMF, and treatment should not be withdrawn abruptly.

**Fig. 6:**
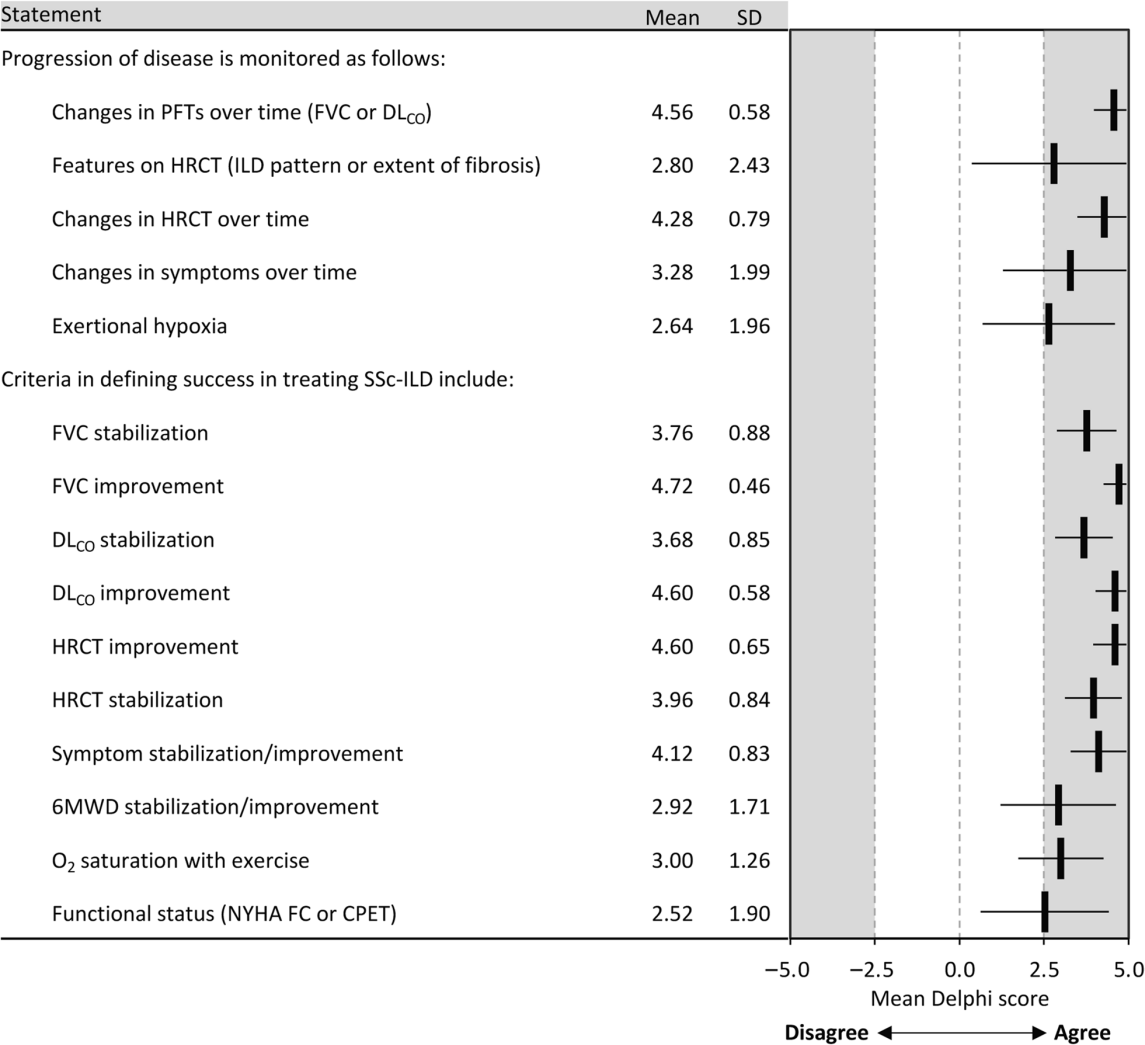
Consensus recommendations for monitoring progression and defining treatment success in SSc-ILD. *6MWD* 6-min walk distance; *CPET* cardiopulmonary exercise testing, *DL*_*CO*_ diffusing capacity of the lungs for carbon monoxide, *FVC* forced vital capacity, *HRCT* high-resolution computed tomography, *ILD* interstitial lung disease, *NYHA FC* New York Heart Association Functional Classification, *PFT* pulmonary function test, *SD* standard deviation, *SSc-ILD* systemic sclerosis-associated ILD

Consensus was reached that disease progression should be monitored using changes in PFTs (FVC or DL_CO_), HRCT (ILD patterns or extent of fibrosis), and symptoms over time, or by presence of desaturation on exercise (Fig. 6). Conversely, stabilization or improvement of FVC, DL_CO_, HRCT, or 6-min walk distance (6MWD), stabilization of symptoms, level of desaturation on exercise, and functional status as assessed by New York Heart Association Functional Classification or cardiopulmonary exercise testing were indicative of treatment success (Fig. 6, Additional file 1: Table S1).

### Management approach based on specialty

There were subtle differences in responses between rheumatologists and pulmonologists (Table 2). Pulmonologists achieved consensus in favor of assessing 6MWD for screening, deciding whether to treat, and defining treatment success, whereas rheumatologists did not rate this assessment so highly. Conversely, pulmonologists did not reach consensus on whether FVC and/or DL_CO_ below the lower limit of normal would prompt immediate treatment. Pulmonologists aligned with panel consensus supporting consideration of reflux when deciding whether to treat; rheumatologists aligned with the panel against the use of methotrexate as an initial treatment option for SSc-ILD. Although both groups agreed that MMF should be the initial treatment of choice, rheumatologists were more comfortable using rituximab as potential therapy.

**Table 2:** Notable differences in ratings between specialties

Statements	Rheumatologists	Pulmonologists
Which of the following would you likely perform to screen the general scleroderma population for ILD?		
6MWD	1.31 (2.69)	2.83 (2.21)
When deciding whether to treat patients for ILD do you consider		
Potential contribution of reflux?	1.92 (1.71)	3.17 (1.19)
In deciding to initiate treatment for SSc-ILD, how important are other parameters besides HRCT and PFTs?		
6MWD	1.39 (1.94)	2.67 (1.50)
“At initial presentation in patients with SSc, this condition would cause me enough concern about near-term ILD that I would start treatment right away”		
FVC and/or DL_CO_ < LLN	3.15 (1.52)	2.25 (2.22)
What initial therapy do you use once you have decided to treat SSc-ILD?		
Methotrexate	–3.31 (2.50)	–2.33 (2.46)
What is your typical/target dose for MMF?		
2000 mg daily	3.08 (0.95)	2.25 (2.42)
What is your typical/target dose for rituximab?		
1 g on days 0 and 15	3.69 (1.70)	0.17 (2.82)
I do not utilize rituximab	–3.31 (2.36)	–2.17 (3.01)
Use of antifibrotic drugs		
I see antifibrotic drugs fitting into the management of SSc-ILD after TCZ*	*2.73 (1.56)*	*1.27 (2.33)*
Use of nintedanib [following publication of SENSCIS and INBUILD trial results]		
Based on lack of effective response or improvement with immunosuppressive agents (MMF/CYC/*TCZ*) as defined by lack of improvement of lung function	1.21 (1.89) 2.09 (2.26)*	2.45 (1.37) 3.00 (0.63)*
Any patient with CTD with clinically significant or worsening ILD	1.64 (1.91) 1.45 (1.97)*	2.36 (1.69) 2.82 (1.25)*
Based on lack of effective response or improvement with immunosuppressive agents (MMF/CYC/*TCZ*) as defined by a combination of no improvement in symptoms; ILD on HRCT; and/or PFTs	2.14 (1.92) 2.55 (2.46)*	3.00 (1.41) 3.55 (0.69)*
Nintedanib fits into the management of SSc-ILD as add-on therapy to TCZ*	*2.64 (1.75)*	*1.73 (2.69)*
Use of TCZ [following publication of focuSSced trial results]*		
*Patients with early SSc and ILD with anti-topoisomerase antibodies*	*2.55 (0.93)*	*2.36 (1.12)*
*Based on active worsening of patient condition as defined by a combination of worsening symptoms; ILD on HRCT; and lung function*	*2.73 (1.95)*	*2.00 (2.49)*
*Based on inability to continue CYC/MMF/antifibrotics due to adverse effects*	*2.91 (0.94)*	*2.27 (2.65)*
*Based on inability to continue CYC/MMF/antifibrotics due to lack of achievement of effective dose with CYC/MMF/antifibrotics*	*2.82 (1.40)*	*2.09 (2.81)*
Based on the following response to SSc-ILD treatment, please indicate your likely course of action:		
With progression/worsening of ILD, I would switch to another agent	3.77 (1.01)	1.42 (3.58)
With progression/worsening of ILD, I would add another agent	3.46 (2.15)	2.33 (3.11)
What circumstances would prompt you to consider weaning a patient from therapy?		
Lack of efficacy	4.23 (1.17)	2.08 (2.64)
How do you wean patients from therapy?		
Stop therapy quickly (over weeks or “cold turkey”)	–4.08 (1.19)	–2.42 (3.26)
What is success to you?		
6MWD stabilization/improvement	2.46 (2.11)	3.42 (1.00)

Results are mean (standard deviation)

*6MWD* 6-min walk distance, *CTD* connective tissue disease, *CYC* cyclophosphamide, *DL*_*CO*_ diffusing capacity of the lungs for carbon monoxide, *FVC* forced vital capacity, *HRCT* high-resolution computed tomography, *ILD* interstitial lung disease, *LLN* lower limit of normal, *MMF* mycophenolate mofetil, *PFT* pulmonary function test, *SSc* systemic sclerosis, *TCZ* tocilizumab

*Data from the 2022 supplementary Delphi

The potential use of antifibrotic drugs was more strongly supported by pulmonologists than by rheumatologists. Pulmonologists (but not rheumatologists) achieved consensus for the use of nintedanib in patients with a lack of response to MMF, CYC, or TCZ as defined by no improvement in lung function, and in any patients with connective tissue disease with clinically significant or worsening ILD. Conversely, there was consensus amongst only rheumatologists that nintedanib could be used as add-on therapy to TCZ.

Overall, rheumatologists were more comfortable than pulmonologists with defining how TCZ should be used as a treatment option for SSc-ILD. There was consensus support amongst rheumatologists (but not pulmonologists) for the use of TCZ in patients with early SSc and ILD with topoisomerase antibodies, worsening condition defined as deteriorating symptoms and ILD on HRCT and PFTs, and those unable to continue CYC, MMF, or antifibrotics due to adverse effects or lack of achievement with the effective dose.

In cases of progression or worsening of ILD, pulmonologists did not achieve consensus on switching to or adding another agent; rheumatologists agreed with both strategies. Regarding weaning, rheumatologists achieved consensus for doing so in cases of lack of efficacy and against stopping therapy quickly; pulmonologists did not achieve consensus on either point.

## Discussion

**Fig. 7:**
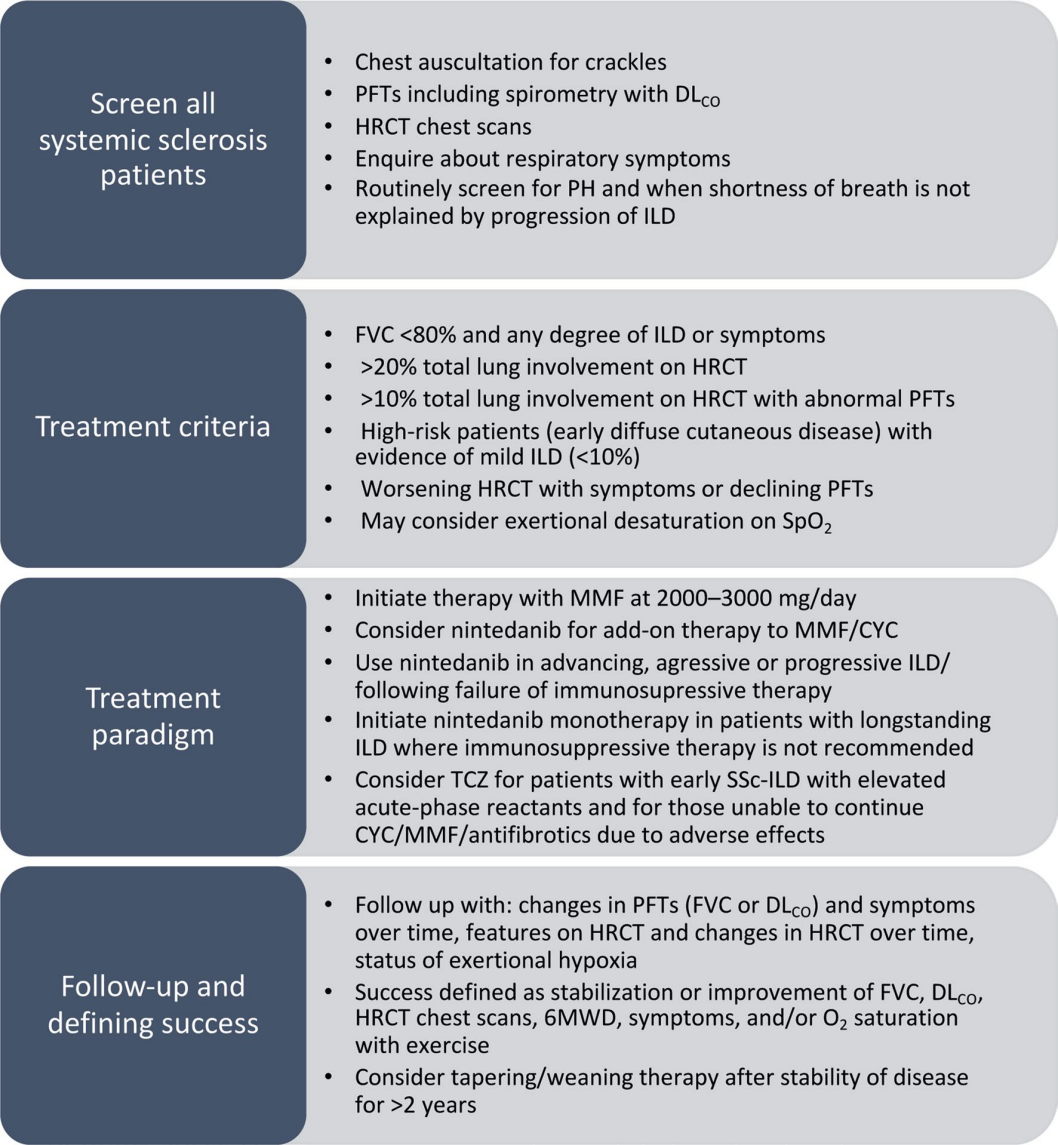
Summary of the consensus recommendations for the management of patients with SSc-ILD. *6MWD* 6-min walk distance, *CYC* cyclophosphamide, *DL*_*CO*_ diffusing capacity of the lungs for carbon monoxide, *FVC* forced vital capacity, *HRCT* high-resolution computed tomography, *ILD* interstitial lung disease, *MMF* mycophenolate mofetil, *PFT* pulmonary function test, *PH* pulmonary hypertension, *SpO*_*2*_ peripheral capillary oxygen saturation, *SSc-ILD* systemic sclerosis-associated ILD, *TCZ* tocilizumab

This Delphi study was initiated to develop consensus recommendations for screening, treatment criteria, and the potential role of antifibrotic drugs in patients with SSc-ILD, building on the latest EULAR scleroderma treatment guidelines and the European consensus statement [12, 14]. The relatively low percentage of statements reaching consensus is reflective of the uncertainty amongst physicians on the appropriate management of SSc-ILD. Nevertheless, the findings from this study provide an algorithm to support effective management of ILD in patients with SSc (Fig. 7), currently the leading cause of death in this population [7, 8].

The expert panelists strongly concurred with the recommendations by the British Society for Rheumatology/British Health Professionals in Rheumatology endorsing ILD screening in all SSc patients [31]. The screening tests recommended through this Delphi process are aligned with published studies defining ILD screening criteria in the general SSc population [1, 4]. However, although HRCT is considered the gold standard for detection of ILD, only 50% of general rheumatologists and 66% of SSc expert rheumatologists order it routinely in newly diagnosed SSc patients, with substantial global practice variation [32].

As well as acknowledging their significance when deciding whether to treat a patient, the panel identified critical thresholds for HRCT, FVC, PFTs, and desaturation on exercise. It is important to note that SpO_2_ should be measured using detection methods such as forehead or ear sensors instead of finger sensors, to avoid misinterpretation resulting from the high prevalence of Raynaud’s phenomenon in this population [33]. Despite the lack of supporting literature, since most randomized controlled trials in SSc-ILD exclude such patients, the panel recommended treating ILD in patients with co-existing PH. Rheumatologists placed more value on autoantibody profiles as a prognostic marker than did pulmonologists, which may reflect differences in familiarity with such biomarkers given the autoimmune nature of many rheumatic conditions. Patients with dcSSc or anti-Scl-70/anti-topoisomerase I antibodies are at higher risk for the development of ILD, whereas patients with limited cutaneous SSc or anti-centromere antibodies are less prone to developing ILD [13]. Consensus was achieved that patients with autoantibodies other than U1 RNP or no specific antibodies should be considered individually to determine the aggressiveness of their disease. There was also consensus that patients with early, rapidly progressive diffuse SSc with mild abnormalities on HRCT and/or PFT would warrant enough concern to support initiating treatment. A broad spectrum of patient characteristics was reviewed when considering who should not be treated for SSc-ILD, with only stable, longstanding disease achieving consensus.

The 2016 EULAR guidelines recommend intravenous CYC for the treatment of SSc-ILD [14]; however, potential toxicity associated with the long-term use of CYC has led to the evaluation of MMF as an alternative. The panel’s near-unanimous endorsement of MMF (with a target of 2000–3000 mg daily) as first-line therapy is supported by the results from the Scleroderma Lung Study II, in which MMF showed a superior safety profile with comparable efficacy to CYC [20]. These results also justify the lack of consensus for the use of CYC as an initial therapy.

Nintedanib (150 mg twice daily) has been approved for the management of patients with SSc-ILD based on results from the SENSCIS trial, which reported a 44% reduction in the annual rate of decline in FVC (the primary endpoint) with nintedanib compared with placebo (P = 0.04) [24]. Following the INBUILD trial, it has also been approved for the treatment of patients with chronic fibrosing ILDs with a progressive phenotype, defined as either an FVC decline of ≥ 10% predicted, or worsening respiratory symptoms and an increased extent of fibrosis on HRCT with or without an FVC decline of 5–10% predicted. The trial demonstrated a 57% reduction in the annual rate of FVC decline with nintedanib compared with placebo (P < 0.001) [25]. Currently, nintedanib is the only antifibrotic drug approved for both indications. In SSc-ILD, the panelists agreed that nintedanib should be considered as add-on therapy to MMF, CYC, or TCZ in patients with advancing or progressive ILD, and after failure of immunosuppressive therapy. In patients with longstanding ILD or in whom immunosuppressive therapy is not recommended or not tolerated, nintedanib may be initiated as monotherapy.

TCZ (162 mg every week) is also approved for the management of pulmonary function decline associated with SSc-ILD. This was based on the results of the focuSSced trial, a multicenter, double-blind, placebo-controlled, phase 3 trial that randomized 210 adults with dcSSc for ≤ 60 months and an mRSS of 10–35 to receive subcutaneous TCZ 162 mg or placebo weekly for 48 weeks in a 1:1 ratio. Although the trial failed to demonstrate a difference in the primary endpoint of mRSS between the groups (P = 0.10), the secondary analysis of FVC and the exploratory analysis of radiographically determined lung fibrosis suggest that TCZ may have the potential to preserve lung function in patients with early diffuse SSc-ILD and elevated acute-phase reactants [29]. There was very little consensus on the use of TCZ as a therapeutic option for patients with SSc-ILD, with greater consensus agreement amongst rheumatologists compared with pulmonologists. This suggests that, since its approval in 2021, pulmonologists are less comfortable than rheumatologists with using TCZ to treat patients with SSc-ILD.

Pirfenidone has been approved for use in patients with IPF, but not in other progressive fibrotic ILDs [34]. The RELIEF trial, a double-blinded, placebo-controlled, prospective phase 2b trial of pirfenidone (2403 mg every day) in patients with fibrotic ILDs other than IPF, was terminated due to futility. However, the results suggest a treatment benefit for patients whose condition deteriorates despite conventional therapy, based on a slower decline of FVC% predicted from baseline compared with placebo [27]. In addition, a trial evaluating the use of pirfenidone in combination with MMF in active and symptomatic SSc-ILD patients is ongoing [28]. These results may further shift our understanding of how antifibrotic drugs can be more appropriately used in the treatment of patients with SSc-ILD.

Predicting the course of SSc-ILD and defining therapeutic goals remain challenging. Panelists agreed that stabilization, as well as improvement, in the signs and symptoms of ILD were indicative of treatment success. Recently, new American Thoracic Society guidelines have placed SSc-ILD within a subgroup of ILDs other than IPF which can manifest progressive pulmonary fibrosis, defining progression as a combination of at least two signs based on symptomological, radiological and physiological findings [35]. For this Delphi, the panel considered changes in FVC, DL_CO_, HRCT, and symptoms over time as key indicators of disease progression, broadly aligned with this guidance as well as previously published reports [1, 4, 25, 35]. The panel was unable to arrive at a consensus on treatment duration. However, they agreed on the conditions under which patients should be weaned off therapy, including weaning over 1–2 years while conducting PFTs every 6 months.

The differences in responses between rheumatologists and pulmonologists tended to reflect practice familiarity. The 6MWD is regularly used in pulmonology, while the use of methotrexate and rituximab is more common in rheumatologic conditions. Data comparing intravenous rituximab with monthly pulses of CYC support the rheumatologists’ recommendation on this point [36]. These differences highlight the importance of multidisciplinary management of SSc-ILD, combining expertise across the multifaceted clinical manifestations of SSc.

There are limitations embedded in the Delphi process. There are no standard criteria defining consensus in Delphi studies, and given the breadth of topics investigated with this method, such standardization may not be feasible. Designed to elicit guidance when no strong evidence is available, the process is not statistically rigorous, and when consensus is reached there is no guarantee that it is generalizable or appropriate. Bias may be introduced during panel selection and development of the initial questionnaire. Anonymity, an integral component of the Delphi process, devolves panelists from accountability for their responses, with the consequence that these may be based on insufficient or minimal consideration. Equal weighting of responses means panelists with relatively less experience may have an impact on consensus disproportionate to their familiarity with the subject matter. In this study, limiting the expert panel to 25 participants to ensure a manageable process may have resulted in missing important perspectives from a larger, more representative population of physicians. Restricting participation to those practicing in the US focused recommendations on those relevant to and feasible within that locale, but limited the capture of global perspectives. Not including patients, pharmacists, payers, and other potential stakeholders (such as physicians practicing in the community) in the panel may have impacted the diversity of opinions and alignment of these recommendations with the Institute for Healthcare Improvement Triple Aim Initiative [37].

## Conclusions

This modified Delphi study involving experts in pulmonology and rheumatology facilitated the development of consensus recommendations and a management algorithm for screening, treatment criteria, and the potential role of antifibrotics and TCZ in patients with SSc-ILD in a real-world setting. Pulmonologists and rheumatologists aligned for the majority of recommendations, with some subtle differences in their perspectives on treatment initiation and therapeutic approaches. These differences highlight the importance of collaborative management of patients and the clinical impact of multidisciplinary discussion groups. Findings from this study provide a management algorithm that will be critical for treating patients with SSc-ILD and help expand on the latest guidelines with clinical expertise and consideration of recently published trials in SSc-ILD.

## Supplementary Information

**Additional file 1. Table S1.** SSc-ILD Delphi Questionnaire 3 results. **Table S2.** SSc-ILD Delphi Supplemental Questionnaire 2 results

## Data Availability

The datasets used and/or analyzed during the current study are available from the corresponding authors on reasonable request.

## Abbreviations

6MWD: 6-Minute walk distance
CYC: Cyclophosphamide
dcSSc: Diffuse cutaneous SSc
DL_CO_: Diffusing capacity of the lungs for carbon monoxide
EULAR: European League Against Rheumatism
FVC: Forced vital capacity
HRCT: High-resolution computed tomography
ILD: Interstitial lung disease
IPF: Idiopathic pulmonary fibrosis
MMF: Mycophenolate mofetil
mRSS: Modified Rodnan skin score
PFT: Pulmonary function test
PH: Pulmonary hypertension
SD: Standard deviation
SpO_2_: Peripheral capillary oxygen desaturation
SSc: Systemic sclerosis
TCZ: Tocilizumab
