# Supplementary material for: Expert consensus on the management of systemic sclerosis-associated interstitial lung disease
Source: Respir Res. 2023 Jan 9;24:6. doi: 10.1186/s12931-022-02292-3 (PMC9830797; doi:10.1186/s12931-022-02292-3)
Supplement: Supplementary file 1 — Additional file 1. Table S1. SSc-ILD Delphi Questionnaire 3 results. Table S2. SSc-ILD Delphi Supplemental Questionnaire 2 results [file 12931_2022_2292_MOESM1_ESM.docx]

**Additional file 1**

**Expert consensus on the management of systemic sclerosis-associated interstitial lung disease**

Franck F. Rahaghi, Vivien M. Hsu, Robert J. Kaner, Maureen D. Mayes, Ivan O. Rosas, Rajan Saggar, Richard M. Silver, Virginia D. Steen, Mary E. Strek, Elana J. Bernstein, Nitin Bhatt,

Flavia V. Castelino, Lorinda Chung, Robyn T. Domsic, Kevin R. Flaherty, Nishant Gupta,

Bashar Kahaleh, Fernando J. Martinez, Lee E. Morrow, Teng Moua, Nina Patel, Oksana A. Shlobin, Brian D. Southern, Elizabeth R. Volkmann, Dinesh Khanna

**Table S1. SSc-ILD Delphi Questionnaire 3 results**

This table contains the questions from Questionnaire 3, the mean and SD of the Likert scale results, and whether consensus was reached or not reached.

| **Statements** | **Mean** | **SD** | **Consensus** |
| --- | --- | --- | --- |
| **How do you screen for SSc-ILD?** | | | |
| **Which of the following would you likely perform to screen the general scleroderma population for ILD?** | | | |
| Spirometry with DL_CO_ | 4.36 | 1.04 | For |
| Full PFT | 4.16 | 1.70 | For |
| Chest x-ray | 0.40 | 3.23 | No |
| HRCT | 4.08 | 1.63 | For |
| Biomarker tests | −0.32 | 2.70 | No |
| Manometry data | −1.92 | 2.14 | No |
| Autoantibody testing | 2.96 | 1.79 | For |
| Echocardiogram | 0.32 | 3.29 | No |
| Doppler | −0.68 | 3.09 | No |
| 6MWD | 2.04 | 2.54 | No |
| Chest auscultation | 4.32 | 1.18 | For |
| **Which patients do you screen for SSc-ILD?** | | | |
| All scleroderma patients | 3.72 | 2.19 | For |
| Patients with symptoms | 4.92 | 0.28 | For |
| High-risk patients (e.g.: dcSSc, +Scl-70 antibodies, African American ethnicity, and/or a high mRSS) | 4.96 | 0.20 | For |
| **Who do you treat?** | | | |
| **When deciding whether to treat patients for ILD do you consider:** | | | |
| Extent of ILD or fibrosis on HRCT | 4.56 | 1.08 | For |
| Autoantibody status | 2.44 | 1.50 | No |
| Baseline PFT values | 4.08 | 0.95 | For |
| Clinically meaningful change in PFT values | 4.76 | 0.52 | For |
| Duration and degree of dyspnea | 3.76 | 1.36 | For |
| Length of disease | 2.36 | 1.38 | No |
| Potential contribution of reflux | 2.52 | 1.58 | For |
| Patient age | 1.28 | 2.46 | No |
| Comorbidities | 2.40 | 1.56 | No |
| Presence of pulmonary hypertension | 3.08 | 1.63 | For |
| **Based on HRCT, do you treat patients who have:** | | | |
| Worsening HRCT with symptoms or declining PFTs | 4.84 | 0.37 | For |
| >20% involvement on HRCT with normal PFTs | 3.04 | 1.57 | For |
| >20% involvement on HRCT with abnormal PFTs | 4.48 | 0.82 | For |
| >10% involvement on HRCT with abnormal PFTs | 3.52 | 1.30 | For |
| High-risk patients (early diffuse disease) with evidence of mild ILD (<10%) and abnormal PFTs | 4.08 | 1.12 | For |
| High-risk patients (early diffuse disease) with evidence of mild ILD (<10%) | 3.24 | 1.51 | For |
| **Based on FVC and symptom status (assume all patients have ILD on HRCT), do you treat patients who have:** | | | |
| FVC >80% with ILD on HRCT in a high-risk patient (early diffuse disease, Topo+) | 3.72 | 1.51 | For |
| FVC >80% with ILD on HRCT in a low-risk patient (early limited disease, centromere+) | 0.72 | 1.93 | No |
| FVC <80% with any degree of ILD on HRCT | 2.68 | 1.80 | For |
| FVC >80% and dyspnea | 2.36 | 1.68 | No |
| FVC <80% and dyspnea | 3.44 | 1.50 | For |
| FVC <70% and dyspnea | 4.12 | 1.24 | For |
| Decline in FVC by greater than measurement error (5–7%) | 4.16 | 0.90 | For |
| Decline in FVC by >10% in 1 year | 4.48 | 0.87 | For |
| Regardless of FVC once other causes of dyspnea are excluded | 2.20 | 1.85 | No |
| **In deciding to initiate treatment for SSc-ILD, how important are other parameters besides HRCT and PFTs?** | | | |
| 6MWD | 2.00 | 1.83 | No |
| Exertional desaturation on SpO_2_ | 3.28 | 1.54 | For |
| Echocardiogram | 1.68 | 2.58 | No |
| Cough consistent with ILD | 1.52 | 1.53 | No |
| Presence of active cutaneous disease | 1.88 | 1.94 | No |
| Presence of reflux | 1.00 | 1.96 | No |
| Scl-70 antibody status | 2.04 | 1.72 | No |
| Elevated BNP | 0.08 | 2.14 | No |
| Evidence of Velcro crackles on lung examination | 2.08 | 1.91 | No |
| **To determine the phenotype of patients that are likely to respond to treatment, which strategy or strategies are you likely to employ?** | | | |
| Autoantibody status | 2.24 | 1.94 | No |
| Cutaneous disease status | 2.28 | 1.88 | No |
| Findings or changes on HRCT | 3.84 | 1.31 | For |
| Findings or changes on PFTs | 3.68 | 1.35 | For |
| Duration of symptoms | 2.72 | 1.54 | For |
| Lung biopsy specimen demonstrating cellular/non-fibrotic NSIP | 1.64 | 2.75 | No |
| Lung biopsy specimen demonstrating UIP | 0.64 | 2.58 | No |
| Concomitant pulmonary vascular disease | 1.20 | 2.35 | No |
| I don’t believe we have enough reliable information at this point to determine the phenotype of patients likely to respond to treatment | 1.48 | 2.55 | No |
| **The following patients should NOT be treated for SSc-ILD:** | | | |
| Preserved PFTs with mild ILD on HRCT | 1.68 | 2.08 | No |
| Patients with history of chronic lung infections, including MAI and aspergillus | 2.16 | 2.27 | No |
| Patients with longstanding disease (close to 10 years) with stable PFTs and no progression of ILD over last few years | 3.52 | 1.81 | For |
| Centromere+ patients | −0.12 | 2.52 | No |
| Stable PFTs over 1 year | 1.32 | 2.14 | No |
| Advanced/end-stage lung disease | 1.84 | 2.61 | No |
| Patients with UIP pattern on HRCT | −0.92 | 2.57 | No |
| Patients who are older | −0.96 | 2.35 | No |
| Patients with significant comorbidities | 0.32 | 1.99 | No |
| Patients with significant recurrent clinical aspiration | 0.28 | 2.07 | No |
| Patients with pulmonary arterial hypertension | −1.44 | 2.22 | No |
| Patients with advanced liver or renal disease who are at risk for more complications related to medical therapies | 1.36 | 2.66 | No |
| **Regarding consideration of autoantibodies in deciding whether to treat patients for SSc-ILD at initial presentation:** | | | |
| I do not consider autoantibodies in deciding whether to treat patients for SSc-ILD | −0.48 | 2.60 | No |
| I consider the presence of anti-Scl-70 and anti-nucleolar pattern on ANA in deciding to treat patients for SSc-ILD | 1.64 | 1.96 | No |
| I consider presence of RNA polymerase III antibodies in deciding to treat patients for SSc-ILD at initial presentation | 1.52 | 2.02 | No |
| Patients with other antibodies than U1 RNP or no specific antibodies have to be taken individually as we don't know how aggressive their disease will be | 2.76 | 2.19 | For |
| In patients with centromere+ antibodies, I am less likely to treat them for SSc-ILD | 0.12 | 2.91 | No |
| In patients with RNA polymerase III+ antibodies, I am less likely to treat them for SSc-ILD | −0.84 | 2.61 | No |
| **“At initial presentation in patients with SSc, this condition would cause me enough concern about near-term ILD that I would start treatment right away”** | | | |
| Moderate-to-severe ILD on HRCT | 4.00 | 1.12 | For |
| FVC and/or DL_CO_ <LLN | 2.72 | 1.90 | For |
| Moderate-to-severe symptoms | 3.68 | 1.07 | For |
| Early, rapidly progressive dcSSc even with mild abnormalities on HRCT chest scan | 3.64 | 1.15 | For |
| Early, rapidly progressive dcSSc even with mild abnormalities on PFT | 3.60 | 1.29 | For |
| Early, rapidly progressive dcSSc even with mild abnormalities on HRCT chest scan AND mild abnormalities on PFT | 4.20 | 0.87 | For |
| Presence of anti-Scl-70 | 1.56 | 1.73 | No |
| HRCT showing ILD >20% lung involvement | 3.80 | 0.96 | For |
| Hypoxemia at rest | 3.92 | 1.12 | For |
| Desaturation on exercise | 3.84 | 1.11 | For |
| **How do you treat?** | | | |
| **What initial therapy do you use once you have decided to treat SSc-ILD?** | | | |
| MMF | 4.72 | 0.74 | For |
| CYC | 0.36 | 2.84 | No |
| Rituximab | 0.52 | 2.66 | No |
| Azathioprine | −0.28 | 2.41 | No |
| Methotrexate | −2.84 | 2.48 | Against |
| Prednisone | −0.60 | 2.75 | No |
| **What is your typical/target dose for MMF?** | | | |
| 1000 mg daily | −2.40 | 2.45 | No |
| 1500 mg daily | −1.88 | 2.67 | No |
| 2000 mg daily | 2.68 | 1.82 | For |
| 3000 mg daily | 4.44 | 0.77 | For |
| I do not utilize MMF | −4.56 | 1.39 | Against |
| **What is your typical/target dose for azathioprine?** | | | |
| 2–3 mg/kg/day | 2.88 | 2.46 | For |
| 50–150 mg/day | 1.04 | 3.34 | No |
| Up to 200 mg/day | 1.04 | 3.39 | No |
| I do not utilize azathioprine | −2.56 | 2.97 | No |
| **What is your typical/target dose for rituximab?** | | | |
| 1 g on days 0 and 15 | 2.00 | 2.89 | No |
| 1 g on days 0 and 15, then every 6–12 months | 2.76 | 2.49 | For |
| 2 g every 6 months | −0.40 | 2.99 | No |
| I do not utilize rituximab | −2.76 | 2.70 | Against |
| **Use of antifibrotic drugs** | | | |
| I see antifibrotic drugs fitting into the management of SSc-ILD after CYC/MMF | 2.36 | 2.46 | No |
| I see antifibrotic drugs fitting into the management of SSc-ILD concomitant to CYC/MMF | 2.96 | 2.37 | For |
| I see antifibrotic drugs fitting into the management of SSc-ILD as determined based on decline in lung function and/or HRCT | 2.64 | 1.96 | For |
| I do not see antifibrotic drugs fitting into the management of SSc-ILD | −4.24 | 1.17 | Against |
| **Use of nintedanib [following publication of SENSCIS trial results]** | | | |
| **I would use nintedanib for treating patients with SSc-ILD under the following clinical conditions:** | | | |
| Patients with progressive fibrotic ILD despite immunosuppressive therapy | 4.28 | 0.79 | For |
| Patients with progressive fibrotic ILD in combination with MMF/CYC | 4.00 | 0.96 | For |
| Patients who have contraindications to or are unable to tolerate immunosuppression | 3.71 | 1.55 | For |
| In combination with immunosuppressive agents (MMF/CYC) for patients with aggressive ILD, advanced disease at initial presentation, or significant disease progression | 3.68 | 1.28 | For |
| Any patient with CTD with clinically significant or worsening ILD | 1.96 | 1.81 | No |
| Based on lack of effective response or improvement with immunosuppressive agents (MMF/CYC) as defined by lack of symptom improvement | 0.96 | 1.65 | No |
| Based on lack of effective response or improvement with immunosuppressive agents (MMF/CYC) as defined by lack of improvement of ILD on HRCT | 1.28 | 1.67 | No |
| Based on lack of effective response or improvement with immunosuppressive agents (MMF/CYC) as defined by lack of improvement of lung function | 1.76 | 1.76 | No |
| Based on lack of effective response or improvement with immunosuppressive agents (MMF/CYC) as defined by a combination of the above | 2.52 | 1.73 | For |
| Based on active worsening of patient condition as defined by worsening of symptoms | 1.76 | 1.42 | No |
| Based on active worsening of patient condition as defined by worsening of ILD on HRCT | 3.00 | 1.29 | For |
| Based on active worsening of patient condition as defined by worsening of lung function | 3.33 | 0.87 | For |
| Based on active worsening of patient condition as defined by a combination of the above | 3.72 | 0.79 | For |
| Based on inability to continue CYC/MMF due to adverse effects | 2.80 | 1.35 | For |
| Based on inability to continue CYC/MMF due to lack of achievement of effective dose with CYC/MMF | 2.00 | 1.53 | No |
| Based on inability to continue CYC/MMF due to prolonged use | 1.21 | 1.93 | No |
| **Nintedanib fits into the management of SSc-ILD as:** | | | |
| Initial monotherapy in cases with significant lung fibrosis (e.g. >10% total lung involvement on HRCT) with preserved FVC and DL_CO_ | −0.88 | 2.13 | No |
| Initial monotherapy in patients with contraindication to or problems tolerating immunosuppressive agents | 1.32 | 2.50 | No |
| Initial monotherapy in patients with >20% total lung involvement on HRCT, indicative of significant fibrosis | 0.04 | 2.14 | No |
| Initial monotherapy in patients with longstanding SSc (>5 years) with ILD and evidence of progression for whom immunosuppression would not be recommended | 2.60 | 1.63 | For |
| Dual initial therapy in combination with MMF/CYC | 0.92 | 2.20 | No |
| Add-on therapy after failure of MMF/CYC | 3.40 | 1.71 | For |
| Add-on therapy to MMF/CYC | 3.36 | 0.91 | For |
| **Based on the following response to SSc-ILD treatment, please indicate your likely course of action:** | | | |
| With progression/worsening of ILD, I would switch to another agent | 2.64 | 2.80 | No |
| With progression/worsening of ILD, I would add another agent | 2.92 | 2.66 | For |
| With progression/worsening of ILD, I would continue treatment as is | −1.44 | 2.84 | No |
| With stability of ILD, I would switch to another agent | −3.12 | 1.88 | Against |
| With stability of ILD, I would add another agent | −3.28 | 1.88 | Against |
| With stability of ILD, I would continue treatment as is | 3.56 | 1.85 | For |
| **How long do you treat?** | | | |
| 2 years | 2.20 | 2.60 | No |
| 5 years | 2.44 | 2.42 | No |
| Continue until stabilization of PFTs and symptoms | 2.28 | 2.94 | No |
| Continue lifelong | 0.28 | 3.39 | No |
| **What circumstances would prompt you to consider weaning a patient from therapy?** | | | |
| Mild-to-moderate disease | 0.56 | 2.52 | No |
| Comorbidities | 2.08 | 1.94 | No |
| Toxicity to the drug (including side effects and adverse events) | 4.08 | 1.00 | For |
| Stability for ≥2 years in lung as well as skin | 3.04 | 2.30 | For |
| Patient's strong desire to discontinue treatment | 3.60 | 1.66 | For |
| Lack of efficacy | 3.20 | 2.26 | For |
| **How do you wean patients from therapy?** | | | |
| Taper/wean over 1–2 years, monitor PFTs every 6 months, with or without low maintenance dose of MMF | 3.56 | 1.66 | For |
| Taper/wean over months to a year to a lower maintenance dose | 2.60 | 1.47 | For |
| Taper/wean over months to a year to off | 1.20 | 2.53 | No |
| Stop therapy quickly (over weeks or “cold turkey”) | −3.28 | 2.51 | Against |
| **What is progression to you, and how do you monitor it?** | | | |
| Absolute values in PFTs (FVC or DL_CO_) | 1.60 | 2.75 | No |
| Changes in PFTs over time (FVC or DL_CO_) | 4.56 | 0.58 | For |
| Features on HRCT (ILD pattern or extent of fibrosis) | 2.80 | 2.43 | For |
| Changes in HRCT over time | 4.28 | 0.79 | For |
| Changes in symptoms over time | 3.28 | 1.99 | For |
| 6MWD | 1.12 | 2.49 | No |
| Patient demographics (age, gender, race) | 0.36 | 2.50 | No |
| Patient autoantibody profile | 0.96 | 2.35 | No |
| Cutaneous disease status/activity | 1.24 | 2.51 | No |
| Exertional hypoxemia | 2.64 | 1.96 | For |
| Development of concomitant GI disease (GERD, microaspiration) | 0.60 | 2.18 | No |
| Development of concomitant pulmonary vascular disease (pulmonary arterial hypertension) | 1.12 | 2.44 | No |
| **What is success to you?** | | | |
| FVC stabilization | 3.76 | 0.88 | For |
| FVC improvement | 4.72 | 0.46 | For |
| DL_CO_ stabilization | 3.68 | 0.85 | For |
| DL_CO_ improvement | 4.60 | 0.58 | For |
| HRCT improvement | 4.60 | 0.65 | For |
| HRCT stabilization | 3.96 | 0.84 | For |
| Symptom stabilization/improvement | 4.12 | 0.83 | For |
| 6MWD stabilization/improvement | 2.92 | 1.71 | For |
| O_2_ saturation with exercise | 3.00 | 1.26 | For |
| mRSS stabilization/improvement | 0.92 | 2.27 | No |
| Functional status (NYHA FC or CPET) | 2.52 | 1.90 | For |

6MWD, 6-minute walk distance; ANA, antinuclear antibodies; BNP, brain natriuretic peptide; CPET, cardiopulmonary exercise testing; CTD, connective tissue disease; CYC, cyclophosphamide; dcSSc, diffuse cutaneous systemic sclerosis; DL_CO_, diffusing capacity of the lungs for carbon monoxide; FCV, forced vital capacity; GERD, gastroesophageal reflux disease; GI, gastrointestinal; HRCT, high-resolution computed tomography; ILD, interstitial lung disease; LLN, lower limit of normal; MAI, *mycobacterium avium-intracellulare*; MMF, mycophenolate mofetil; mRSS, modified Rodnan skin score; NSIP, non-specific interstitial pneumonia; NYHA FC, New York Heart Association Functional Classification; PFT, pulmonary function test; RNP, ribonucleoprotein; SD, standard deviation; SpO_2_, peripheral capillary oxygen saturation; SSc-ILD, systemic sclerosis-associated ILD; TCZ, tocilizumab; Topo, topoisomerase; UIP, usual interstitial pneumonia.

**Table S2. SSc-ILD Delphi Supplemental Questionnaire 2 results**

This table contains the questions from Supplemental Questionnaire 2, the mean and SD of the Likert scale results, and whether consensus was reached or not reached.

| **Statements** | **Mean** | **SD** | **Consensus** |
| --- | --- | --- | --- |
| **How do you treat?** | | | |
| **Use of antifibrotic drugs** | | | |
| I see antifibrotic drugs fitting into the management of SSc-ILD after CYC/MMF | 3.91 | 0.61 | For |
| I see antifibrotic drugs fitting into the management of SSc-ILD concomitant to CYC/MMF | 3.00 | 1.07 | For |
| I see antifibrotic drugs fitting into the management of SSc-ILD after TCZ | 2.00 | 2.07 | No |
| I see antifibrotic drugs fitting into the management of SSc-ILD concomitant to TCZ | 0.68 | 2.36 | No |
| I see antifibrotic drugs fitting into the management of SSc-ILD before TCZ | 0.27 | 2.49 | No |
| I see antifibrotic drugs fitting into the management of SSc-ILD as determined based on decline in lung function and/or HRCT | 3.82 | 0.91 | For |
| I do not see antifibrotic drugs fitting into the management of SSc-ILD | -4.68 | 0.57 | Against |
| **Use of nintedanib** | | | |
| **I would use nintedanib for treating patients with SSc-ILD under the following clinical conditions:** | | | |
| Patients with progressive fibrotic ILD despite immunosuppressive therapy (MMF/CYC/TCZ) | 4.55 | 0.80 | For |
| Patients with progressive fibrotic ILD in combination with MMF/CYC/TCZ | 4.23 | 0.87 | For |
| Patients who have contraindications to or are unable to tolerate immunosuppression (MMF/CYC/TCZ) | 4.32 | 0.65 | For |
| In combination with immunosuppressive agents (MMF/CYC/TCZ) for patients with aggressive ILD, advanced disease at initial presentation, or significant disease progression | 3.95 | 0.84 | For |
| Any patient with CTD with clinically significant or worsening ILD | 2.14 | 1.75 | No |
| Based on lack of effective response or improvement with immunosuppressive agents (MMF/CYC/TCZ) as defined by lack of symptom improvement | 2.05 | 0.95 | No |
| Based on lack of effective response or improvement with immunosuppressive agents (MMF/CYC/TCZ) as defined by lack of improvement of ILD on HRCT | 1.82 | 1.71 | No |
| Based on lack of effective response or improvement with immunosuppressive agents (MMF/CYC/TCZ) as defined by lack of improvement of lung function | 2.55 | 1.68 | For |
| Based on lack of effective response or improvement with immunosuppressive agents (MMF/CYC/TCZ) as defined by a combination of the above | 3.05 | 1.84 | For |
| Based on active worsening of patient condition as defined by worsening of symptoms | 2.14 | 1.08 | No |
| Based on active worsening of patient condition as defined by worsening of ILD on HRCT | 3.59 | 0.67 | For |
| Based on active worsening of patient condition as defined by worsening of lung function | 3.86 | 0.56 | For |
| Based on active worsening of patient condition as defined by a combination of the above | 4.05 | 0.49 | For |
| Based on inability to continue CYC/MMF/TCZ due to adverse effects | 3.14 | 0.94 | For |
| Based on inability to continue CYC/MMF/TCZ due to lack of achievement of effective dose with CYC/MMF/TCZ | 2.68 | 0.99 | For |
| Based on inability to continue CYC/MMF/TCZ due to prolonged use | 1.18 | 1.82 | No |
| **Nintedanib fits into the management of SSc-ILD as:** | | | |
| Initial monotherapy in cases with significant lung fibrosis (e.g. >10% total lung involvement on HRCT) with preserved FVC and DL_CO_ | –1.55 | 1.22 | No |
| Initial monotherapy in patients with contraindication to or problems tolerating immunosuppressive agents | 2.27 | 1.80 | No |
| Initial monotherapy in patients with >20% total lung involvement on HRCT, indicative of significant fibrosis | 0.59 | 2.26 | No |
| Initial monotherapy in patients with longstanding SSc (>5 years) with ILD and evidence of progression for whom immunosuppression would not be recommended | 3.05 | 1.21 | For |
| Dual initial therapy in combination with MMF/CYC | 1.59 | 1.50 | No |
| Dual initial therapy in combination with TCZ | -0.05 | 2.19 | No |
| Add-on therapy after failure of MMF | 3.82 | 0.66 | For |
| Add-on therapy after failure of CYC | 3.64 | 0.79 | For |
| Add-on therapy after failure of TCZ | 3.00 | 1.93 | For |
| Add-on therapy after failure of MMF and CYC | 3.82 | 0.73 | For |
| Add-on therapy after failure of TCZ and CYC | 3.59 | 0.73 | For |
| Add-on therapy after failure of TCZ and MMF | 3.64 | 0.66 | For |
| Add-on therapy after failure of TCZ and  MMF and CYC | 3.64 | 0.58 | For |
| Add-on therapy to MMF/CYC | 3.77 | 0.81 | For |
| Add-on therapy to TCZ | 2.18 | 2.26 | No |
| **Use of TCZ** | | | |
| **I would use TCZ for treating patients with SSc-ILD under the following  clinical conditions:** | | | |
| Patients with early SSc and ILD with progressive skin disease | 1.45 | 2.13 | No |
| Patients with early SSc and ILD with anti-topoisomerase antibodies | 2.45 | 1.01 | No |
| Patients with early SSc and ILD with elevated acute-phase reactants | 3.18 | 0.96 | For |
| Any patient with early SSc and ILD | –0.23 | 2.18 | No |
| Patients with clinical ILD (according to symptoms, HRCT, FVC and/or DL_CO_) with active extrapulmonary manifestations | 0.32 | 2.10 | No |
| Patients with clinical ILD (according to symptoms, HRCT, FVC and/or DL_CO_) with quiescent extrapulmonary manifestations | 0.77 | 2.02 | No |
| Patients with progressive ILD despite antifibrotic therapy | 1.14 | 2.27 | No |
| Patients with progressive ILD despite immunosuppressive therapy (MMF/CYC) | 1.59 | 2.17 | No |
| Patients with progressive ILD in combination with MMF/CYC | 0.18 | 2.02 | No |
| Patients with progressive ILD in combination with antifibrotics | 1.82 | 2.63 | No |
| Patients who have contraindications to or are unable to tolerate immunosuppression (CYC/MMF) | 1.95 | 2.10 | No |
| Patients who have contraindications to or are unable to tolerate antifibrotics | 2.00 | 2.43 | No |
| In combination with immunosuppressive agents (MMF/CYC) for patients with aggressive ILD, advanced disease at initial presentation, or significant disease progression | 0.68 | 2.19 | No |
| In combination with antifibrotics for patients with aggressive ILD, advanced disease at initial presentation, or significant disease progression | 1.55 | 2.11 | No |
| Any patient with CTD with clinically significant or worsening ILD | –0.50 | 2.06 | No |
| Based on lack of effective response or improvement with immunosuppressive agents (MMF/CYC) or antifibrotics as defined by lack of symptom improvement | 0.64 | 2.13 | No |
| Based on lack of effective response or improvement with immunosuppressive agents (MMF/CYC) or antifibrotics as defined by lack of improvement of ILD on HRCT | 1.14 | 2.27 | No |
| Based on lack of effective response or improvement with immunosuppressive agents (MMF/CYC) or antifibrotics as defined by lack of improvement of lung function | 1.50 | 2.37 | No |
| Based on lack of effective response or improvement with immunosuppressive agents (MMF/CYC) or antifibrotics as defined by a combination of the above | 1.82 | 2.36 | No |
| Based on active worsening of patient condition as defined by worsening of symptoms | 0.73 | 1.83 | No |
| Based on active worsening of patient condition as defined by worsening of ILD on HRCT | 1.64 | 2.32 | No |
| Based on active worsening of patient condition as defined by worsening of lung function | 2.23 | 2.18 | No |
| Based on active worsening of patient condition as defined by a combination of the above | 2.36 | 2.22 | No |
| Based on inability to continue CYC/MMF/antifibrotics due to adverse effects | 2.59 | 1.97 | For |
| Based on inability to continue CYC/MMF/antifibrotics due to lack of achievement of effective dose with CYC/MMF/antifibrotics | 2.45 | 2.20 | No |
| Based on inability to continue CYC/MMF/antifibrotics due to prolonged use | 1.09 | 2.02 | No |
| **TCZ fits into the management of SSc-ILD as:** | | | |
| Initial monotherapy in patients with contraindication to or problems tolerating antifibrotics | –0.36 | 2.04 | No |
| Initial monotherapy in patients with contraindication to or problems tolerating immunosuppressive agents (CYC/MMF) | 1.68 | 2.03 | No |
| Initial monotherapy in patients with any SSc-ILD | –1.09 | 2.20 | No |
| Initial monotherapy in patients with early SSc and ILD | –0.05 | 2.08 | No |
| Dual initial therapy in combination with antifibrotics | –0.32 | 2.10 | No |
| Dual initial therapy in combination with MMF/CYC | –0.68 | 1.76 | No |
| Add-on therapy after failure of MMF as initial therapy, before changing MMF to CYC | 1.95 | 1.89 | No |
| Replacement therapy following failure of MMF as initial therapy, before changing to CYC | 2.14 | 2.03 | No |
| Add-on therapy after failure of CYC as initial therapy | 0.45 | 2.09 | No |
| Add-on therapy after failure of antifibrotics | 0.91 | 2.07 | No |
| Add-on therapy after failure of MMF and CYC | 1.64 | 1.97 | No |
| Add-on therapy after failure of MMF and antifibrotics | 1.64 | 1.97 | No |
| Add-on therapy after failure of CYC and antifibrotics | 1.32 | 1.99 | No |
| Add-on therapy after failure of MMF and CYC and antifibrotics | 1.59 | 2.13 | No |
| Add-on therapy to MMF/CYC | 0.91 | 1.87 | No |
| Add-on therapy to antifibrotics | 1.36 | 2.08 | No |
| **What is your typical/target dose for nintedanib?** | | | |
| I do not utilize nintedanib | –4.73 | 0.55 | Against |
| 100 mg twice daily | 0.50 | 2.24 | No |
| 150 mg twice daily | 4.68 | 0.57 | For |
| **What is your typical/target dose for TCZ?** | | | |
| I do not utilize TCZ | –3.36 | 1.89 | Against |
| 162 mg/week | 4.18 | 1.44 | For |
| **When do you screen for pulmonary hypertension in patients with SSc-ILD?** | | | |
| I do not screen for pulmonary hypertension | –4.77 | 0.53 | Against |
| At every patient consultation | –0.14 | 2.51 | No |
| Once per year | 4.41 | 0.85 | For |
| Every 2 years | 0.32 | 2.48 | No |
| I routinely screen for pulmonary hypertension | 4.59 | 0.73 | For |
| I screen for pulmonary hypertension in patients aged >50 years | 2.00 | 2.29 | No |
| I screen for pulmonary hypertension when shortness of breath is not explained by progression of ILD | 4.86 | 0.47 | For |

CTD, connective tissue disease; CYC, cyclophosphamide; DLCO, diffusing capacity of the lungs for carbon monoxide; FCV, forced vital capacity; HRCT, high-resolution computed tomography; ILD, interstitial lung disease; MMF, mycophenolate mofetil; SD, standard deviation; SSc, systemic sclerosis; TCZ, tocilizumab.
